# Degos disease with multiple intestinal perforations: A missed-opportunity case report and literature review

**DOI:** 10.3389/fcvm.2022.910288

**Published:** 2022-10-17

**Authors:** Wen Ai, Zhihua Liang, Feng Li, Haihua Yu

**Affiliations:** Department of Gastrointestinal Surgery, The First Affiliated Hospital of Shandong First Medical University, Jinan, China

**Keywords:** Degos disease, malignant atrophic papulosis, intestinal perforations, skin eruption, case report

## Abstract

**Introduction:**

Degos disease, also known as malignant atrophic papulosis (MAP), is a rare systemic obstructive vascular disease with unknown pathophysiology, which can affect multiple systems, especially gastrointestinal tract and central nervous system. Intestinal perforations with MAP is associated with high mortality rate and ambiguous treatment outcomes.

**Case presentation:**

Here we report a missed-opportunity case of Degos disease characterized by generalized skin eruption and multiple intestinal perforations. Definite diagnosis of Degos disease was finally concluded after two exploratory laparotomy operations and skin biopsies. Due to the delayed diagnosis and treatment, the patient died after being discharged automatically in spite of application of aspirin and low-dose subcutaneous heparin. In view of such circumstances, we searched the Pubmed using “Degos [Title] OR Malignant Atrophic Papulosis [Title]” AND “perforation [Title] OR perforations [Title]” and make a detailed analysis of the result.

**Conclusions:**

Degos disease is a rare systemic obstructive vascular disease with unknown pathologic mechanism and unavailable treatment methods. Diagnosis is usually based on the presence of pathognomonic skin lesions and tissue biopsy. Gastrointestinal involvement can cause serious and lethal conditions with high mortality. Currently, how to achieve a satisfying prognosis of MAP with intestinal perforations becomes the most urgent problem in front of medical staff.

## Introduction

Degos disease, also known as malignant atrophic papulosis (MAP), is a rare systemic obstructive vascular disease with unknown pathophysiology, which can affect multiple systems, mainly involving gastrointestinal tract and central nervous system, leading to high mortality (1). Here we report a missed-opportunity case of Degos disease characterized by generalized skin eruption and multiple intestinal perforations and make a literature review of other analogous cases.

## Case presentation

**Figure 1:**
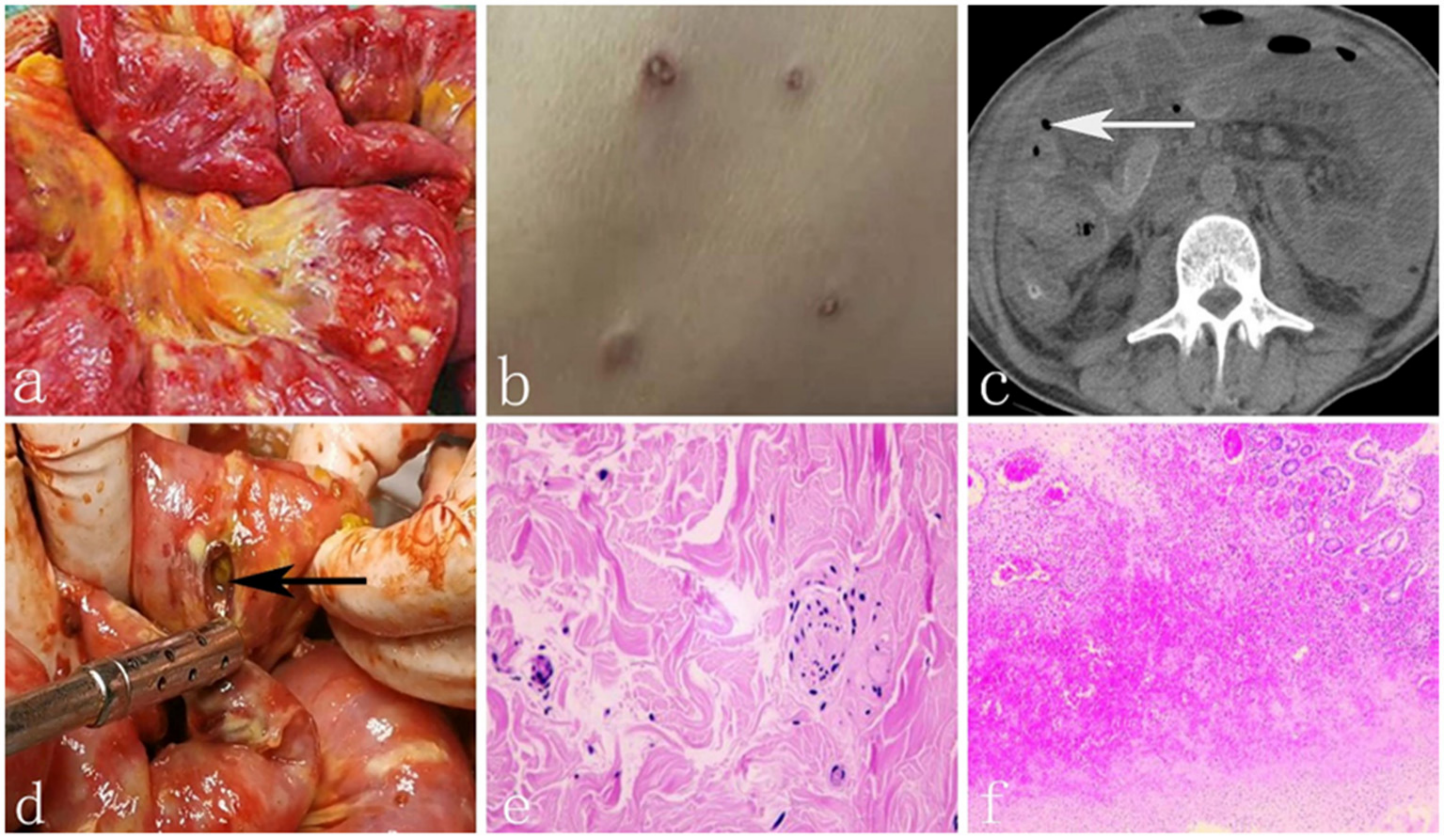
**(a)** Numerous yellow-white tubercles were interspersed on the surface of small intestine. **(b)** Generalized skin emption with porcelain - white centers surrounded by erythematous borders. **(c)** The white arrow shows free air around the intestine. **(d)** The black arrow shows one of the perforations of the small intestine. **(e)** The biopsy of skin eruption showed atrophy of epidermis, hyperplasia and collagen of dermal fibrous tissue. **(f)** Histopathologic result of the resected intestine showed acute and chronic inflammatory cell infiltration.

A 48-year-old man was admitted to our hospital's emergency department with a 2-month history of recurrent abdominal pain and a 2-week history of aggravation. He underwent an exploratory laparotomy for acute diffuse peritonitis 50 days ago at the local hospital. Intraoperative findings revealed that numerous 0.2–0.4 cm yellow-white tubercles were interspersed on the surface of congestive small intestine, colon, appendix, greater omentum and mesenterium, especially the distal ileum (Figure 1a), without apparent perforation. Rapid pathology of appendix and omentum showed acute nonspecific inflammation and intestinal tuberculosis was suspected initially. Therefore large amount of saline and metronidazole was used for irrigation and abdominal drainage was placed. The patient discharged from hospital with intermittent slight abdominal pain on the 15th day after operation.

Twenty days later, the patient developed severe abdominal pain suddenly with nausea and vomiting accompanied by fever (39°C). He was admitted to the local hospital again and transferred to our intensive care unit (ICU) for deterioration of his condition. Physical examination showed evident abdominal tenderness and rebound tenderness with weakened bowel sounds. Meanwhile, generalized skin eruptions with an atrophic porcelain-white center surrounded by erythematous rim were visible over trunk and extremities, measuring 0.2–1.2 cm (Figure 1b). No chronic diseases, no alcohol use, no family history, no herbal agents, or no suspected drug use were reported. Relevant laboratory tests and radiographic results were as follows: WBC 8.65^*^10^9^ (3.5–9.5^*^10^9^), NEUT% 0.938↑ (0.40–0.75), procalcitonin 171.45 (0–0.05 ng/ml), HBV-DNA 9.15E+08↑ (<5.0E+02 IU/ml). Tuberculin test, Widder test, anti SS-A, and SS-B antibodies were negative. ANA was 1:100 weakly positive and Anti Ro-52 antibodies was positive too. Computed tomography showed a small amount of free air bubble, edema and thickening of the small bowel wall and massive seroperitoneum (Figure 1c). An exploratory laparotomy was performed on account of primary diagnosis of Crohn's disease with intestinal perforations. In the operation, we found more than 100 whitish-yellow plaques, dozens of intestinal perforations and purulent materials (Figure 1d). We resected 2 meters of the perforated small bowel and sutured the seromuscular layer in the wafery areas followed by enterostomy and abdominal drainage tube placement. The diagnosis of Crohn's disease was doubtful for the eccentric appearance of the intestine conditions, but there was still no definite diagnosis.

On the 5th day after operation, intestinal contents drained again, which signified recurrence of perforation. At this point, we eventually started to pay attention to the correlation between the perforation and skin lesions. Biopsy of skin eruption was conducted and the pathology showed atrophy of epidermis, hyperplasia and collagen of dermal fibrous tissue, degeneration of elastic fibers, significantly reduction and partial necrosis of skin appendages, fibrinoid necrosis in small vessels of the deep dermis and thrombosis in local lumen (Figure 1e). Histopathology of the resected intestine showed edema and hyperaemia, thrombus organization of both tiny artery and veins lumen with acute and chronic inflammatory cell infiltration (Figure 1f). Finally, definite diagnosis of Degos disease was concluded. The patient was given aspirin (100 mg/day) and low-dose subcutaneous heparin (5,000 U/dy), but his body condition went from bad to worse because of the severe abdominal inafection. He requested automatic discharge and died 3 days later.

## Discussion and conclusions

Malignant atrophic papulosis (MAP) was first described by Köhlmeier in 1941 (2) and defined by Degos in 1942 (3), which usually occurs in the 20–50 age group with a slight male dominance (4). Previous appellation way of MAP was chaotic until Theodoridis et al. (5) renamed and divided it into two categories in 2014: benign atrophic papulosis (BAP) and malignant atrophic papulosis (MAP). The former is more common and characterized by cutaneous form, with atrophic porcelain-white center in size of 0.2–1 cm, distributed over the trunk and extremities, rarely on the face and scalp. The papules are red in early stage and expand gradually with atrophy in the center and leave white scars after deflorescence. The latter is characterized by involvement of internal organs, especially gastrointestinal tract (50%) and central nerves system (20%) (1). Median survival time of MAP is 2–3 years and five-year survival rate is less than 50% (6). However, these two forms can't be easily distinguished because involvement of inner organs may occur with skin lesions simultaneously or not (7).

Intestinal perforations with MAP is an uncommon phenomenon with a rate of 2.1% reported (8). We searched the Pubmed using “Degos [Title] OR Malignant Atrophic Papulosis [Title]” AND “perforation[Title] OR perforations[Title]” and found 11 cases. Nevertheless, the detailed data of 1 case reported by G H Evans (9) couldn't be enquired, so only the 10 cases were exhibited in Table 1 (10–19). As shown in the literature, the median age was 41.5 (range 7–56) with a male 60% proportion and only three of them were alive at follow-up. Table 2 showed four case study series ever published (5, 18, 20, 21). The age at diagnosis ranges from 34 to 37.9 years old and MAP accounts for 65% of the total cases (155:239). Two studies (5, 18) reported 0% mortality and another (20)reported 3% mortality for BAP while the mortality for MAP is 65.3–75%. Given MAP's high mortality rate, Theodoridis et al. (5) put forward a follow-up plan: whole-skin examination and skin biopsy for histological examination for BAP and colonoscopy/gastroscopy/laparoscopy if organ symptomatology suspected, the follow-up frequency was twice yearly for 0–7 years and once yearly for 7–10 years.

**Table 1:** Previous case reports of MAP and intestinal perforations.

**Author/year**	**Age/sex**	**Number of intestinal perforations**	**Surgery (yes or no)**	**Operation frequency**	**Operation method**	**Postoperative treatment**	**Prognosis**	**Survival period or follow-up period**
M Kanai/1988 (10**)**	47M	3	Yes	1	Enterectomy	Not mentioned	Alive	10 months
FMG Valverde/2003 (11**)**	56M	1/multilple necrotic lesions	Yes	2	Perforation repair	Not mentioned	Death	Several days
Shahshahani MM/2008 (12**)**	47F	1	Yes	1	Enterectomy	Antiplatelet/anticoagulant/ pentoxifylline	Death	A few months
M Beuran/2009 (13**)**	29F	2/not mentioned/not mentioned/not mentioned	Yes	4	Not mentioned	Not mentioned	Death	3 months
XY Zheng/2010 (14**)**	37M	diffuse plaque lesions/2	Yes	2	Omentectomy/ Jejunostomy	Methylprednisolone/ immunoglobulin/ anticoagulation	Death	3 months
Ahmadi/2011 (15**)**	15F	2/1/2	Yes	3	Enterectomy/Ileostomy/ Right hemicolectomy	Aspirin/Dipyridamol	Death	3 months
Yeung/2013 (16**)**	50M	Multiple perforations/Multiple perforations/Multiple perforations	Yes	3	Enterectomy	Palliative care	Death	Not mentioned
Zhu/2014 (17**)**	46F	1	Yes	1	Enterectomy	Heparin heparin/Cefodizime/dipyridamole	Alive	7 years and 10 months
Hu/2018 (18**)**	30M	Not mentioned	No	No	No	Bayaspirin/Dalteparin sodium/alprostadil/methylprednisolone	Death	Not mentioned
Day/2019 (19**)**	7M	Not mentioned	Yes	multiple surgeries	Not mentioned	Cyclophosphamide/rituximab	Alive	23 months
Present case	48M	>10	Yes	1	Entetectomy and jejunostomy	Aspirin/heparin	Death	16 days

**Table 2:** Demographic and clinical data of four previous studies.

	**Burg/** **1989 (20)**	**Assier/** **1995 (21)**	**Theodoridis/** **2014 (5)**	**Hu/** **2018 (18)**
Study cohort size	106	15	39	80
Male/female (ratio)	63:43	6:9	16:23	37:43
Age at diagnosis	35	34	36.5	37.9
BAP/MAP (ratio)	39:67	6:9	11:27	28:52
Total mortality	51/106 (48.1)	Not mentioned	8/38 (21%)	34/80 (42.5%)
BAP mortality	1/39 (3%)	Not mentioned	0/27 (0%)	0/28 (0%)
MAP mortality	50/67(75%)	Not mentioned	8/11(73%)	34/52 (65.3%)
Survival time with systemic presentation	<5 years	Not mentioned	0.9 year	Not mentioned

Etiology of Degos disease remains unknown up to now. Viral infection, autoimmune disease, coagulopathy, collagen vascular disorders and genetic defects may be some of the underlying causes (12). In this case, the level of patient's HBV-DNA may be an explanation of MAP since it is 9.15E+08↑ IU/ml, significantly higher than normal. Autosomal dominant trait of MAP is hypothesized in view of reports of clusters of patients among members of the same family and first-degree relatives (22). Diagnosis of MAP depends mainly on presence of pathognomonic skin lesions and tissue biopsy pathology containing wedge necrosis of superficial and deep dermis, with arteriolar wall inflammatory cell infiltration, epidermal atrophy and dermal collagen rigidification. Dermoscopy reveals dendritic vessels, loop and irregular vessels, with central unstructured area but it lacks specificity (23).

At present, there is still no clear guideline for the treatment of MAP. Immuno-suppressor, such as azathioprine and cyclophosphamide are proved to be invalid (24). Antiplatelet drugs (aspirin or clopidogrel) or anticoagulants (warfarin or heparin) may be helpful (25). In 2011 Magro CM reported the pathologic findings of extensive deposits of C5b−9 within the cutaneous vasculature, and proposed that inhibition of C5 might be a therapeutic approach (26), and in 2013 he discovered the use of eculizumab as salvage therapy in critically ill patients with thrombotic micro angiopathy (27). Shapiro LS thought that treprostinil may offer a second effective treatment approach to individuals with MAP or rescue therapy to those in whom eculizumab treatment has failed (28). Unfortunately, large sample data results are still lacking, especially for gastrointestinal tract perforations.

In conclusion, Degos disease is a rare systemic obstructive vascular disease with unknown pathologic mechanism and unavailable treatment methods. Diagnosis is usually based on the presence of the pathognomonic skin lesions and tissue biopsy. Gastrointestinal involvement can cause serious and lethal conditions with high mortality (29).The patient's diagnosis was delayed because we ignored the relation between skin changes and peritonitis. Although current pharmacological treatments have limited value for MAP with intestinal perforations, early diagnosis and treatment play an important role in improving prognosis and survival rate.

## Data availability statement

The original contributions presented in the study are included in the article/supplementary material, further inquiries can be directed to the corresponding author.

## Ethics statement

Written informed consent was obtained from the participant/s for the publication of this case report. Written informed consent was obtained from the individual(s), and minor(s)' legal guardian/next of kin, for the publication of any potentially identifiable images or data included in this article.

## Author contributions

WA wrote the manuscript. ZL and FL searched literatures. HY revised and approved the final manuscript. All authors have read and approved the manuscript.

## Conflict of interest

The authors declare that the research was conducted in the absence of any commercial or financial relationships that could be construed as a potential conflict of interest.

## Publisher's note

All claims expressed in this article are solely those of the authors and do not necessarily represent those of their affiliated organizations, or those of the publisher, the editors and the reviewers. Any product that may be evaluated in this article, or claim that may be made by its manufacturer, is not guaranteed or endorsed by the publisher.

## Abbreviations

MAP: malignant atrophic papulosis
BAP: benign atrophic papulosis
ICU: intensive care unit
WBC: white blood cell
NEUT: neutrophil
HBV-DNA: hepatitis B virus-DNA
ANA: antinuclear antibodies.
